# Access to Reproductive Health Services and Maternal Perceptions on Family Planning in an Indigenous Guatemalan Valley

**DOI:** 10.1155/2018/7879230

**Published:** 2018-12-31

**Authors:** Lauren Ashley Lambert, Jeremy Brittingham Hatcher, Xinyu Wang

**Affiliations:** ^1^Department of Spanish and Portuguese, Vanderbilt University, PMB 401617 2301 Vanderbilt Place Nashville, TN 37240, USA; ^2^Vanderbilt University Medical Center, 1211 Medical Center Dr. Nashville, TN 37232, USA; ^3^Department of Statistics, University of Virginia, PO Box 400135, 148 Amphitheater Way, Charlottesville, VA 22903, USA

## Abstract

*Objective. *To identify reproductive health barriers and perceptions regarding family planning among mothers in ten rural communities of Guatemala.* Methods. *Data were collected from 85 women in a Nutrition Recuperation Project (NRP) conducted by a freestanding nonprofit clinic in Palajunoj Valley, Guatemala. All nonpregnant women participating in the NRP were eligible for enrollment in this study, and NRP staff members aided in their enrollment. Participants were interviewed and data were entered into a structured questionnaire. Data analysis was conducted using R version 1.1.456.* Results. *After asking participants if they believed fertility is higher on certain days, only 5 women (5.9%) correctly identified these days as occurring in the middle of the menstrual cycle. 35 women (41.2%) practiced some form of family planning, and 27 (31.8%) reported that they do not know of a place where they could obtain a contraceptive method.* Conclusion. *There is a lack of education regarding family planning methods in this valley, and the levels of contraception use are below average for rural Guatemala. These findings may implicate substantial health risks for women and children in the valley, and they support the pertinence of education-based interventions in the area of reproductive health behaviors.

## 1. Introduction

According to a 2015 USAID Report, Guatemala has the highest under-5 child mortality rate in all of Central America [1]. In a data analysis of 52 developing countries, it was found that under-5 child deaths would fall by 13 percent if interpregnancy intervals were at least 24 months [2]. Since high total fertility rates (TFRs) are associated with short interpregnancy intervals, Guatemala's high TFR is likely a large contributor to the under-5 child mortality rate [2]. Further, data suggests that there is a disparity between Guatemalan rural and urban TFRs. The rural Guatemalan TFR of 4.2 far outpaces the urban TFR of 2.9 [1]. Concentrated poverty in rural communities also creates issues including lack of access to healthcare infrastructure and proper nutrition [3]. Guatemala's high rate of malnutrition [4] and low use of modern contraceptives [1] are exacerbated among rural and indigenous populations. Further, previous findings show that out of all Guatemalan population groups, intent to use contraception rates are lowest for indigenous women [5]. Thus, there is a harmful combination of high TFR and low health-promoting resources in rural Guatemalan communities. Importantly, this theme in rural health is also observed globally. Even in countries where the majority of the population lives in rural areas, the majority of the healthcare resources are located in the cities [6]. Additionally, WHO reports state that 214 million women in developing countries who want to avoid pregnancy are not using family planning as a result of limited access to contraception, which is particularly seen among poorer population groups [7].

Primeros Pasos is a nonprofit clinic located in Guatemala, a country in which 75.7% of the indigenous are poor and 70.5% of the rural residents are poor [8]. The clinic is located in the Palajunoj Valley, where 92.7% of the population lives in rural areas and 95% identify as indigenous Maya. Home to 14,481 inhabitants, the valley consists of the ten communities: Xecaracoj, Chuicaracoj, Chuicavioc, Las Majadas, Tierra Colorada Alta, Tierra Colorada Baja, Xepaché, Candelaria, Bella Vista, and Llano del Pinal [9]. In response to a lack of maternal healthcare and nutrition resources offered in the valley, Primeros Pasos launched a Nutrition Recuperation Program (NRP) for mothers in the surrounding communities in 2013. Guatemalan nutritionists visit all 10 communities each week in order to monitor the height and weight of the enrolled women's children as well as conduct workshops on nutrition topics. While this program previously focused primarily on the malnutrition issue in the valley, the directors suspected the need for an expanded education-based intervention encompassing reproductive health topics. The aim of the current study was to aid the clinic's efforts and collect information regarding the current reproductive health barriers experienced by women in the valley. This study formally investigated mothers' perceptions on and needs related to such topics, and in late 2017 the clinic implemented curriculum changes to address the needs identified by this study.

## 2. Materials and Methods

Prior to this study, all mothers living in the Palajunoj Valley were given the opportunity to participate in Primeros Pasos' Nutrition Recuperation Program (NRP), which supports chronically malnourished children and women through medical interventions and health education. This study was conducted 6 months into the NRP, and all nonpregnant women enrolled in the NRP were eligible to participate in the study. The study was conducted over a two-week period in July 2017. Interviews were conducted at Primeros Pasos after women obtained an annual free health consult. Pregnant women were excluded due to their potentially vulnerable status. Since clinic directors have found that many women who are pregnant in these communities have high levels of anxiety regarding pregnancy outcomes, the directors did not want the survey to perpetuate any negative self-directed emotions that women may have been experiencing following unwanted pregnancies. Ethical approval was obtained by the Institutional Review Board at Vanderbilt University.

The women that agreed to participate were interviewed using appropriate questions taken from the “Encuesta Nacional de Salud (Materno Infantil)” (National Survey of Maternal and Children's Health), which was conducted by the DHS (Demographic and Health Services Survey) Program in Guatemala in 2014 and 2015 [10]. The survey consisted of 62 qualitative questions, 3 quantitative questions, and 5 open ended questions. The survey was comprised of the following sections: “Basic Questions,” “Family Planning,” “Childbirth/Children,” “Sexually Transmitted Diseases,” and “Gynecological Services.” The data were input in a structured questionnaire, and all the interviews were conducted in Spanish. Each woman was interviewed privately within the clinic with only the principal investigator present. Potential participants were asked if they would like to sign a consent document. If the participant did not agree to signing the consent document, they were given the opportunity to provide verbal consent. No identifiable collected patient information was shared with Primeros Pasos, nor any other health center.

Data were exported to Excel 2016 and analyzed in R version 1.1.456. Based on a population size of 14,481, the necessary sample size was calculated to be 67 when assuming a 90% confidence interval. 85 women participated in the study. Chi-squared tests for independence were conducted in order to analyze responses, and* P*<0.1 was considered statistically significant. Additionally, the associated Cramer's V value was calculated for each chi-squared test in order to measure the strength of the association. Descriptive statistics were reported as frequencies and percentages with 90% confidence intervals.

## 3. Results

Overall, 85 out of 123 women gave consent and participated in the study (response rate 69.1%). The mean age was 28.1±1.28 with a median age of 27, indicating only a slight skewness in the data towards older women. 16 women (18.8%) reported that they could not read, and 11 women (12.9%) reported that they could read “un poquito” (a little).

The mean age of first childbirth was 19.8±0.7, and the mean number of children per mother was 2.9±0.39. Of all reported births recorded in this study, 42.9% were delivered in a home with a “comadrona” (midwife) present. 38 women (44.7%) wanted to have another child in the future, and 35 (92.1%) of those women wanted to wait prior to having that child. The mean number of years that the women wanted to wait was 4.3±0.61, but only 16 (42.1%) of those women were using some form of contraception (defined as using any of the methods in Table 3). Thus, the majority of these women are at high risk of unwanted pregnancy. Additionally, a chi-squared test for independence found no statistically significant association between the desire to wait prior to having another child and contraceptive use (*χ*^2^=0.9395,* P*=0.3324, df=1), and a Cramer's V value of 0.1594 further confirmed that the association is weak (Table 1). However, the small sample size of women poses a risk to the reliability of this finding.

Among all 85 women, 35 (41.2%) were using some form of contraception at the time of the survey. For those using a family planning method, 10 (28.6%) reported having received the female sterilization procedure, making this the most common form of contraception. Each participant was also asked about the types of family planning methods they had heard of. An extensive list (Table 2) was read, and after hearing descriptions for each method (Table 3), participants provided yes/no responses. The most frequently reported form of known contraception was “Injections” (71, 83.5%) (Table 2). However, more than a quarter of all women (27, 31.8%) reported that they did not know of a place where they could obtain a contraceptive method. A chi-squared test also revealed that there was no association between one's distance to the closest city (Quetzaltenango) and contraceptive use (*χ*^2^=2.0196,* P*=0.3643, df=2). The associated Cramer's V value of 0.1541 confirmed the weak association (Table 1). Additional chi-squared tests for independence were performed to determine if knowledge about certain forms of contraception was associated with contraceptive use. An association was found between active contraceptive use and knowledge of male sterilization (*χ*^2^=4.82,* P*=0.03), knowledge of IUDs (*χ*^2^=11.06,* P*=0.0009), knowledge about injections (*χ*^2^=6.42,* P*=0.01), and knowledge about the necklace method (*χ*^2^=4.82,* P*=0.03)(all with df=1). The respective Cramer's V values were 0.24, 0.36, 0.27, and 0.24, meaning that the associations were moderate to very strong (Table 1).

When asked about whether there are certain days between menstruations in which it is easier to get pregnant, 58 women (68.2%) reported they believed these fertile days exist. Women that reported “yes” or “maybe” to this question (63 in total) were then asked when these fertile days occur (immediately before or after one's menstrual cycle, in the middle of one's menstrual cycle, or during menstruation). Of the 63 women, only 5 correctly identified these days as occurring in the middle of one's menstrual cycle. Therefore, only 5.9% of the total participants were aware of fertile days and could correctly identify when they occur.

The participants were also asked about whether they had heard of sexually transmitted diseases (STDs) and/or uterine cancer (Table 4). A statistically significant association was found between whether women had heard of STDs and whether she or her partner was using a contraceptive method (*χ*^2^=4.0537,* P*=0.0441, df=1). The associated Cramer's V value was found to be 0.2237, suggesting that the level of association between the two variables is moderate. Women using a contraceptive method were more likely to respond “yes” when asked if they had heard of STDs compared to women who were not using contraception.

**Figure 1:**
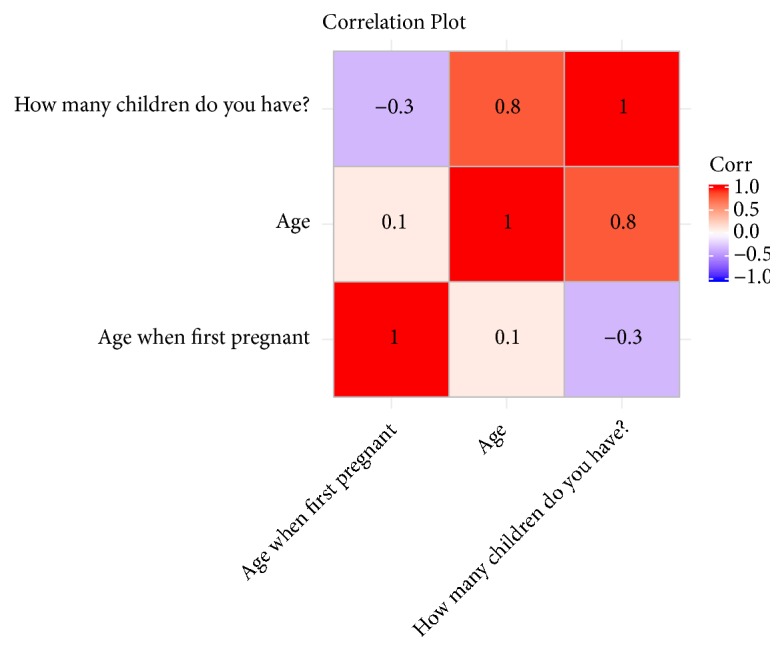
Correlation plot of relationships between age and number of children.

In order to better understand the fertility rate in the Palajunoj Valley, a correlation matrix was utilized to determine the relationship between the number of children women have and their age when first pregnant. The three numerical variables used were age at time of survey, age when first pregnant, and number of children (Figure 1). The matrix revealed a strong positive correlation between age at time of survey and the number of children. Additionally, a moderately weak negative correlation was found between the age when first pregnant and the number of children.

## 4. Discussion

Latin American indigenous women are disproportionally affected by reproductive health barriers and unwanted pregnancies, with a major contributing factor being substandard family planning services [12]. Guatemala's 2014-2015 national study of maternal and infantile health found that indigenous women are less likely to use a method of contraception (50.1% indigenous versus 67.8% nonindigenous) [10]. In the present study, only 35 women (41.2%) were using a family planning method. This contraception utilization rate is lower than the national rural rate (55.3%) and much lower than the national urban rate (68.2%) [10]. Furthermore, the most common form of contraception in the valley was female sterilization, with rates of nonsterilization contraceptive use at only 30.6% compared to the national rate of 39.6% [10]. This suggests that the majority of women in this valley are not using contraception while they are still having children. It is well-studied that rural Latin American communities with high TFRs and low family planning method availability and utilization experience higher rates of adverse health outcomes for both the mother and children, including a higher under-5 child mortality rate [2]. Short interpregnancy intervals have also been found to increase the risk of stillbirth, early neonatal death, and preterm birth [13]. Overall, the findings of the current study indicate that contraceptive and child-spacing practices in this valley are well below the national average for rural populations.

In terms of understanding factors that may contribute to these findings, the study found an association between knowledge of STDs and the use of a contraceptive method. Knowledge of specific types of contraceptives (male sterilization, IUDs, injections, and the necklace method) was also associated with the use of contraceptives. In the opinion of the authors, this is an indicator that educational interventions related to women's health could possibly increase the prevalence of family planning practices in the valley. Furthermore, the moderately weak negative correlation between age when first pregnant and number of children suggests that earlier use of family planning practices might decrease the rate of childbearing. Notably, no association was found to exist between the desire to space future pregnancies and contraceptive use. Also of note was the lack of association between contraceptive use and distance from the nearest city. However, this finding is consistent with previous findings that rural residence is not a barrier to contraceptive use among indigenous women [14]. Factors that have been shown to increase indigenous women's contraceptive use include higher levels of education and greater household wealth [15]. Among 52 countries that have had at least one Demographic and Health Survey since 2001, the most common reason for nonuse of contraceptives is health concern regarding possible negative side effects [16]. Together, these findings support the impetus for education-based interventions.

Education gaps regarding STDs, uterine cancer, and the ovulation process were identified in this study. Out of 85 participants, 30 women (35.3%) had not heard of STDs and 29 women (34.1%) had not heard of uterine cancer. Only 5 women (5.9%) correctly identified when fertile days occur. This is an area for further future research, as the author postulates that women in this valley and similar indigenous communities in Latin America who have less knowledge of STDs and/or uterine cancer will be less likely to seek out or utilize available screening methods.

A limitation of this study is that the data is all self-reported, and recall bias could limit the power of this study to provide accurate statistics regarding health behaviors and community health metrics. Additionally, the sample size was small compared to the total number of women in the valley and the confidence interval of 90% is relatively large. This study also did not examine confounding variables that may affect and help explain the relationships discovered between variables including STD knowledge and contraceptive use. It is possible that factors including income and relative education level could explain some of the associations found between the variables in this study. Further research could potentially clarify the role of these factors in the particular context of the valley. Finally, it is possible that the women in the study have perspectives on healthcare that differ from women who are pregnant or who elected not to participate in the NRP. Since no reproductive health research had previously been conducted in the valley, a strength of this study is the novelty of the topic in the region.

It is noteworthy that this study takes place within a specific regional and cultural context, and study findings are specific to these communities. While the authors make no claims that the study findings can be generalized to other rural regions in Central America, this study shows nuanced community patterns that fit into the general picture of health outcomes and driving sociocultural factors in rural Guatemala and, thus, may provide insight for future investigations. On an international level, this study supports previous findings that reveal a global unmet need for contraception that is concentrated in poor, indigenous, and rural regions of developing countries [7].

Since all participants in the current study were enrolled in NRP executed by Primeros Pasos, this nonprofit has the ability to provide education-based interventions in order to alleviate the educational gaps identified in the present study. The NRP has already effectively created a safe space and women's forum for the indigenous women living in the valley. Similarly, a maternity waiting home called “Casa Materna” (Mother's House) is located in Huehuetenango, Guatemala, and is executing a similar intervention involving women's support groups (WSGs) for indigenous women. These WSGs have enhanced the participating women's self-esteem, which was shown to be associated with women's decisions to seek care [17]. Additionally, evidence suggests that indigenous Guatemalans often seek healthcare with nongovernmental organizations (NGOs) rather than the Ministry of Health because of poor patient satisfaction with the quality of care offered at the institutional level [18]. Such factors have given Primeros Pasos a unique opportunity to meet the needs of the women in the valley. However, the success of NGO efforts is contingent upon whether local knowledge, perceptions, and self-identified needs are taken into account when executing interventions [12].

## 5. Conclusions

The present study found a widespread lack of education regarding fertility, family planning methods, and local reproductive health resources in the Palajunoj Valley. Based on these findings, the author created a reproductive health curriculum (located in “Supplementary Materials” (available here)) to be included in the NRP conducted by Primeros Pasos. The curriculum is now a fully integrated part of the NRP and is being taught to all ten communities in the valley. Specifically, the curriculum focuses on forms of contraception, the ovulation process, STDs, and uterine cancer. Future research should be conducted on women's perceptions on the topics. The author also believes surveys similar to the one used in the present study could identify concordant patterns of low reproductive health education throughout rural indigenous communities in Guatemala. Further, the success (or lack thereof) of the Primeros Pasos community-tailored reproductive education curriculum could be evaluated as an intervention to reduce unwanted pregnancy and high child mortality.

## Figures and Tables

**Table 1:** Interpretation of Cramer's V value [11].

Level of Association	Verbal Description
0.00	No Relationship
0.00 to 0.15	Very Weak
0.15 to 0.20	Weak
0.20 to 0.25	Moderate
0.25 to 0.30	Moderately Strong
0.30 to 0.35	Strong
0.35 to 0.40	Very Strong
0.40 to 0.50	Worrisomely Strong
0.50 to 0.99	Redundant
1.00	Perfect Relationship

**Table 2:** Knowledge of family planning/contraception types (n=85).

Form of contraception	No. (%, 90% confidence interval)
Female sterilization	61 (71.8, 63.7-79.8)
Male sterilization	45 (52.9, 44.0-61.8)
IUD	54 (63.5, 54.9-72.1)
Injections	71 (83.5, 76.9-90.1)
Implant	42 (49.4, 40.5-58.3)
Birth control pills	68 (80.0, 72.9-87.1)
Condom	67 (78.8, 71.5-86.1)
Female condom	31 (36.5, 27.9-45.1)
Vaginal methods	26 (30.6, 22.4-38.8)
Lactation and amenorrhea methods	39 (45.9, 37.0-54.8)
Necklace method	45 (52.9, 44.0-61.8)
Withdrawal	26 (30.6, 22.4-38.8)
Emergency contraception	17 (20.0, 12.9-27.1)

**Table 3:** Descriptions provided for each form of contraception  ^a^.

Form of contraception	Description provided
Female sterilization	When women undergo surgery to avoid having more children
Male sterilization	When men undergo surgery to prevent a woman from getting pregnant
IUD	Women may ask a doctor or nurse to put a spiral, ring, or copper T in the uterus.
Injections	Women may ask a health worker to give them an injection to avoid getting pregnant for one or more months.
Implant	Women may ask a doctor or nurse to place capsules (tubes) under their arm skin to avoid getting pregnant for one or several years.
Birth control pills	Women can take a pill every day to avoid getting pregnant.
Condom	Men can put a rubber band or latex on the penis during sex.
Female condom	Women can put a rubber band on their vagina before sexual intercourse.
Vaginal methods	Women may place a cream, a diaphragm, or tablets inside the vagina before having intercourse.
Lactation and amenorrhea methods	The woman can delay her period after delivery by breastfeeding exclusively day and night when the baby is less than 6 months old.
Necklace method	To avoid getting pregnant, women can count the days of their menstrual cycle with the beads of a necklace and avoid sexual intercourse on days when the beads are white.
Withdrawal	Men can be careful and withdraw before terminating the sexual act.
Emergency contraception	As an emergency measure, within three days of having sex, women may take special pills to keep them from getting pregnant.

**Table 4:** Knowledge of sexual transmitted diseases and uterine cancer (n=85). ^a^

	Yes	No	Unsure
Heard of sexually transmitted diseases	55 (64.7)	30 (35.3)	0
Heard of HIV	62 (72.9)	23 (27.1)	0
Heard of AIDS	69 (81.2)	16 (18.8)	0
Heard of uterine cancer	55 (64.7)	29 (34.1)	1 (1.2)
Heard of a Pap smear	59 (69.4)	26 (30.6)	0
Have had a Pap smear	38 (44.7)	47 (55.3)	0

a: values are given as number (percentage).

## Data Availability

The questionnaire data used to support the findings of this study are restricted by the Institutional Review Board at Vanderbilt University in order to protect patient privacy. Data are available from Lauren Lambert (lauren.a.lambert@vanderbilt.edu) for researchers who meet the criteria for access to confidential data.

## Supplementary Materials

Supplementary Materials The supplementary file contains the reproductive health curriculum (in Spanish) that the Primeros Pasos clinic implemented as a result of this study. Guatemalan medical students teach this curriculum during women's health workshops in the ten rural communities of the Palajunoj Valley.
