# Supplementary material for: Access to Reproductive Health Services and Maternal Perceptions on Family Planning in an Indigenous Guatemalan Valley
Source: Int J Reprod Med. 2018 Dec 31;2018:7879230. doi: 10.1155/2018/7879230 (PMC6332965; doi:10.1155/2018/7879230)
Supplement: Supplementary Materials — The supplementary file contains the reproductive health curriculum (in Spanish) that the Primeros Pasos clinic implemented as a result of this study. Guatemalan medical students teach this curriculum during women's health workshops in the ten rural communities of the Palajunoj Valley. [file 7879230.f1.docx]

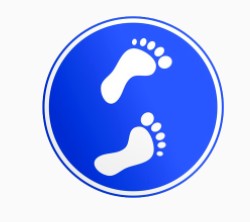


Nuevo material para charlas reproductivas Primeros Pasos

El programa de nutrición Por: Lauren Lambert

El julio, 2017


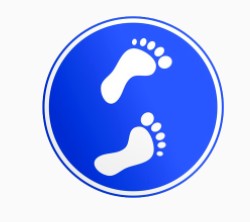


Tabla de contenidos

La investigación con el programa de nutrición… 3

El nivel de la educación de las mujeres 4

[La ovulación… 5-7](#_TOC_250005)

[Los partos por cesárea… 8-9](#_TOC_250004)

[Los métodos de la planificación familiar… 10-17](#_TOC_250003)

[Las enfermedades transmitidas por relaciones sexuales 18-20](#_TOC_250002)

[El cáncer de la matriz 21](#_TOC_250001)

[Referencias 22-23](#_TOC_250000)

La investigación (a través de una encuesta) con 85 mujeres en el programa de nutrición

## Un resumen: ¿Qué hicimos?

Bethan Crisp y Lauren Lambert hicieron una encuesta por dos semanas con las mujeres en el programa de nutrición. La ejecutaron cuando las mujeres vinieron para las jornadas médicas. Las directoras (Scarlet y Monica) ayudaron mucho y aprobaron las preguntas en la encuesta. La meta de este estudio fue ayudar a Scarlet y Monica, las mujeres en el programa de nutrición y la clínica de Primeros Pasos. Después de la investigación, Bethan y Lauren identificaron muchas necesidades con respeto a la planificación familiar, los partos, la educación sobre la salud de las mujeres (enfermedades sexuales, ovulación, etc.) y las enfermedades culturales (el chipe, mal de ojo, etc.). Bethan ha creado un documento con más información sobre las enfermedades culturales para que los empleados de Primeros Pasos puedan entender y respetar las creencias de las mujeres. Por favor, consulte estos documentos (en Google Drive) para aprender más sobre estos temas. En este documento, hay más información y un currículo educativo acerca del resto de los temas en nuestra investigación. Con este material, los directores pueden crear charlas y proveer más educación diseñada especialmente para las mujeres alrededor de la clínica. Lauren ha buscado todo el material en este documento e incluido una lista de referencias al final. Si alguien tiene una pregunta sobre el trabajo y los resultados, contacte a Lauren (correo: [lauren.a.lambert@vanderbilt.edu](mailto:lauren.a.lambert@vanderbilt.edu) o lauren.lambert81@gmail.com). Por último, hay un documento en Google Drive con todas las estadísticas concretas. El propósito de este documento es resumir los resultados de la encuesta y proveer recursos para aliviar las necesidades identificadas.

## Los temas en la encuesta

- El nivel de estudios (de las mujeres)
- El embarazo y el parto (las experiencias y cuidado médico)
- La planificación familiar (el uso y las opiniones)
- Los servicios ginecológicos
- Las enfermedades culturales (mal de ojo, lombrices, chipe, etc.) (OJO: los documentos por Bethan Crisp – no hay información sobre este tema en este documento)

El nivel de educación de las mujeres

| **Nivel** | **Porcentaje** |
| --- | --- |
| Pre-­primaria/nunca estudió | 20.23% |
| Primaria | 60.71% |
| Secundaria | 15.48% |
| Más | 3.57% |

Más estadísticas:

- 19% no puede leer
- 13% puede leer un poquito
- 21% no puede escribir
- 11% puede escribir un poquito

## ¿Por qué es importante?

Muchas mujeres dijeron que los niños están aprendiendo sobre el embarazo, la ovulación, etc. en las escuelas. Sin embargo, la mayoría de estas mujeres no han recibido esta educación. Por eso, es probable que el programa de nutrición sea la única manera en la que las mujeres puedan exponerse a y aprender sobre estos temas. En este documento, hay cajas con recomendaciones abajo para charlas posibles en el futuro.

***(OJO: Los finales de las paginas tienen estas recomendaciones. Se puede referir a la página (la que provee la información y educación) para planear las charlas).***

# La ovulación

## ¿Qué piensan las mujeres sobre los días fértiles?

Pregunta #1 en la encuesta: “Entre una menstruación y otra ¿cree usted que hay ciertos días en los que una mujer puede quedar más fácilmente embarazada si tiene relaciones sexuales? “

Respuestas (en porcentajes de lo que las mujeres dijeron):

- Sí: 68.24%
- No: 14.12%
- Tal vez: 4.71%
- No sabe: 12.94%

Pregunta #2 en la encuesta: “En su opinión, cuáles son esos días ¿antes de que comience la menstruación, durante la menstruación, en la mitad del ciclo menstrual o inmediatamente después de terminar la menstruación?”

Respuestas (en porcentajes de lo que las mujeres dijeron):

- Justo antes que comience la menstruación: 17.46%
- Durante la menstruación: 7.94%
- Inmediatamente después de terminar la menstruación: 41.27% **(La respuesta más común)**
- En la mitad del ciclo menstrual: 7.94% **(La respuesta correcta)**
- Otro: 9.53%
- No sabe: 15.87%

**Conclusión:** La mayoría de las mujeres en el programa no sabe las fechas correctas.


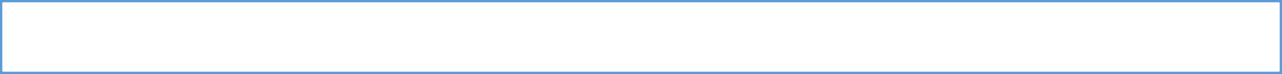


**Matterial para enseñar en una charla:**

1. ¿Qué es ovulación?

El cerebro suelta una hormona llamada LH (hormona lutenizing) y causa la liberación de un óvulo hasta el ovario. Al mismo tiempo, la mucosidad cervical se convierte en una mucosidad resbaladiza. Este evento deja que la esperma alcance el óvulo. Las mujeres nacen con 1-­2 millón óvulos;; ellas sueltan un óvulo por mes hasta menopausia. Cuando el ovario suelta el óvulo, el óvulo viaja por las trompas de Falopio (las que conectan el ovario al útero). Una mujer puede quedar embarazada si la esperma se encuentra el óvulo durante cuyo viaje al útero (causando la fecundación). Si la esperma no se lo encuentra, el óvulo disuelve después de 1-­2 días. La mujer no puede quedar embarazada

si el ovario no ha soltado un óvulo. Sin embargo, la ovulación puede ocurre durante tiempos diferentes para cada mujer. Por eso, es necesario que las mujeres conozcan sus cuerpos, los síntomas y la gama de las fechas durante las que la ovulación puede ocurrir. En resumen, la ovulación es el único tiempo cuando una mujer pueda quedar embarazada.


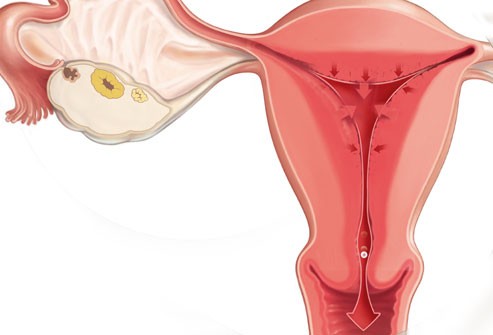

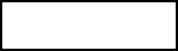

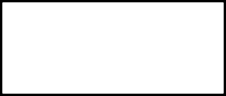

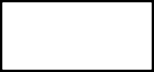


El útero

El ovario

Las trompas de Falopio

1. ¿Cuándo ocurre?

La ovulación ocurre entre los días 11 y 21 del ciclo menstrual. El ciclo menstrual empieza el primer día de la menstruación (el primer día con sangramiento) y termina el día antes de la próxima menstruación. Es decir, la fecha de ovulación depende de cuando la mujer termina la menstruación y su propio cuerpo. El promedio número de días en el ciclo menstrual es 28-­35 días. La época con la probabilidad más alta de quedar embarazada toma lugar durante los dos días antes de que comience la ovulación. Por eso, las mujeres deben aprender a identificar cuando la ovulación ocurre y evitar tener sexo por dos días antes (si ellas no quieren quedar embarazada) (“Fertility & Ovulation Pictures: Facts to Help You Get Pregnant”, *WebMD.com*).

Todas las mujeres tienen la ovulación en diferentes tiempos. Para identificar cuando ocurre, consulte esta lista de síntomas (“Signs and Symptoms of Ovulation”, *Americanpregnancy.org*):

- - El flujo cervical cambia: hay más flujo blanco con una textura gruesa
  - La temperatura del cuerpo cambia: la temperatura baja justo antes de que comience la ovulación y aumenta mucho inmediatamente después (se puede monitorear eso con un termómetro).
  - La sensibilidad de los pechos
  - Más deseo de tener sexo
  - Algunos calambres

El ciclo menstrual:

| Menstruación  Días 1-­5 (aproximadamente) | Días interinos  Días 6-­9 (aproximadamente) | Días **más** fértiles – 2 días antes de ovulación  Días 10-­11 (aproximadamente) | La ovulación (días fértiles)  Entre los días 11-­21 | Días interinos  Días 21-­  28 | Menstruación  Día 1 |
| --- | --- | --- | --- | --- | --- |

Links a la información:

- [http://www.webmd.com/baby/ss/slideshow-­](http://www.webmd.com/baby/ss/slideshow-)understanding-­fertility-­ovulation
- [http://americanpregnancy.org/getting-­](http://americanpregnancy.org/getting-)pregnant/signs-­of-­ovulation/

# Los partos por cesárea

## Las estadísticas sobre las tasas de partos por cesárea:

- En todo el país de Guatemala: 26% (“Delivery Care”, *UNICEF.org*)
- En las comunidades alrededor de Primeros Pasos: 38% (De acuerdo con nuestra encuesta)
- La tasa recomendable: 10-­15% (“WHO Statement on Cesarean Section Rates”,

*WHO.int*)

- Las tasas de partos por cesárea en Latinoamérica y el Caribe son las más altas del mundo (Betrán et al., “The Increasing Trend in Ceasarean Section Rates: Global, Regional and National Estimates: 1990-­2014”)

## ¿Por qué hay bastante partos por cesárea?

- El doctor puede programar el parto (para tener un horario concreto), el parto es más rápido (no necesita esperar por dilatación y contracciones), el hospital puede cobrar más por esta operación y el doctor puede tener un horario más fácil con los partos por cesárea (Khazan, “Why Most Brazilian Women Get C-­Sections”)
- Algunas mujeres prefieren un parto por cesárea (De acuerdo con nuestra encuesta)


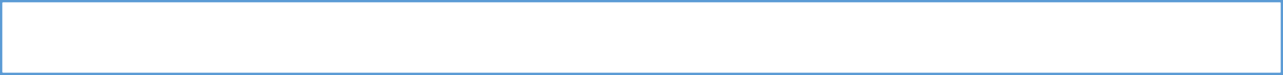


**Matterial para enseñar en una charla:**

1. ¿Cuáles son los riesgos con un parto por cesárea?

De acuerdo con un artículo llamado “Cesarean Section – Risks and Complications” en el sitio *WebMD.com*, los riesgos incluyen:

- Más tiempo para recuperar (en comparación con el período de recuperación con un parto natural)
- La infección
- La pérdida de sangre (hemorragia)
- La coagulación en las piernas o en los pulmones
- La nausea
- El vómito
- Las jaquecas
- El estreñimiento
- La muerte (este riesgo es raro)
- La rotura de la incisión en partos futuros
- La placenta previa (cuando la placenta crece bajo el útero y bloquea el cérvix)

1. ¿Cuándo es necesario tener un parto por cesárea?

Es importante reconocer que hay situaciones en las que un parto por cesárea es necesario. De acuerdo con el mismo artículo (notado arriba) de *WebMD.com*, las razones por un parto por cesárea incluyen:

- El bebé no está en la posición correcta o el bebé está sentado (cuando la cabeza no está abajo)
- Cuando no hay suministro suficiente de sangre en la placenta
- Cuando el bebé tiene un peso de más de 9 libras
- Cuando la madre tiene una enfermedad seria y el parto pueda causar más problemas
- Cuando el bebé tiene una enfermedad (como espina bífida)
- La placenta previa (cuando la placenta crece bajo el útero y bloquea el cérvix)
- Las enfermedades transmitidas por relaciones sexuales que puedan infectar al bebé
- Algunas situaciones con gemelos

Estas complicaciones pueden requerir un parto por cesárea. Sin embargo, si la madre (o el bebé) no está consciente de estas complicaciones, un parto por cesárea puede causar daño. Es una operación con riesgos, y por eso es esencial que la mujer hable con un médico sobre las razones específicas por un parto de cesárea.

Links a la información:

- https://data.unicef.org/topic/maternal-­health/delivery-­care/
- [http://www.who.int/reproductivehealth/publications/maternal_perinatal_health/cs-­](http://www.who.int/reproductivehealth/publications/maternal_perinatal_health/cs-) statement/en/
- https:[//www.ncbi.nlm.nih.gov/pmc/articles/PMC4743929/](http://www.ncbi.nlm.nih.gov/pmc/articles/PMC4743929/)
- https:[//www.theatlantic.com/health/archive/2014/04/why-­](http://www.theatlantic.com/health/archive/2014/04/why-)most-­brazilian-­women-­ get-­c-­sections/360589/
- [http://www.webmd.com/baby/tc/cesarean-­](http://www.webmd.com/baby/tc/cesarean-)section-­risks-­and-­complications

# Los métodos de la planificación familiar

## Las estadísticas importantes (de la encuesta) sobre la planificación familiar:

- Preguntamos sobre los tipos de planificación familiar en los que las mujeres estaban familiarizadas. Solamente 31% de las mujeres están familiarizadas con “Retiro”. Nosotros explicamos el significado de “Retiro” con el siguiente: “Retiro es cuando los hombres pueden tener cuidado y retirarse antes de terminar el acto sexual”.
- 59% de las mujeres no están usando la planificación familiar.
- De las mujeres que están planeando, el método más común es: Esterilización femenina (la operación). 28% de las mujeres han tenido esta operación. (Es decir, las mujeres no están planeando entre los hijos. La mayoría de las mujeres están dando a luz a todos los hijos y después ellas tienen la operación para evitar tener más hijos. Con problemas económicos y una falta de recursos, puede ser beneficioso planear antes de que ellas decidan que no quieran más hijos).
- 31% de las mujeres no conocen algún lugar donde puedan obtener una herramienta de la planificación familiar.
- Solamente 8% de las mujeres mencionaron Primeros Pasos como un lugar donde puedan obtener un método de la planificación familiar.
- 71% de las mujeres quieren aprender más sobre la planificación familiar.
- 48% de las mujeres creen dicen que usarán algún método de la planificación familiar en el futuro.
- 91% de las mujeres que quieren tener otro hijo en el futuro quieren esperar antes de tener este hijo. El promedio número de años que ellas quieren esperar:

4.3 años. (¡Sin embargo, es imposible hacer esto sin la planificación!)

## Como resultado de estos resultados, es necesario enseñar sobre:

- La importancia de usar la planificación familiar
- El método de retiro (Este método es lo más fácil para evitar tener más hijos. Con el retiro, no es probable que la esperma alcance el óvulo).
- Los lugares que ofrecen la planificación familiar
- Las ofertas de Primeros Pasos

**(OJO: Se puede enseñarlas en una charla. Consulte “Material para enseñar en una charla” abajo para más información sobre estos temas).**

## ¿Qué piensan las mujeres sobre este tema?

Pregunta #1 en la encuesta: Si la mujer dijo que ella no usó la planificación familiar, la preguntamos: “¿Por qué no usa la planificación familiar?”

Respuestas (en porcentajes de lo que las mujeres dijeron):

- 20% dijo que no sabe porque no usa
- 11% dijo que tiene miedo de los efectos secundarios
- 8% dijo la razón sencilla: “No quiero usarlo”
- 7% dijo que el esposo no la permite

Pregunta #2 en la encuesta: Si la mujer dijo que ella querría aprender sobre la planificación familiar, la preguntamos: “¿Qué quiere aprender sobre la planificación familiar?”

Respuestas reales y más comunes:

- Las opciones y métodos diferentes
- El valor y derecho de la mujer
- Donde se puede aprender y obtener los métodos
- Cualquier cosa sobre la planificación familiar
- Cual método funciona mejor
- Sobre los métodos naturales
- Sobre los mitos de planificación familiar
- Sobre las enfermedades que una mujer puede tener e infecciones vaginales

**(OJO: hay información sobre el último tema^ en la página 17)**


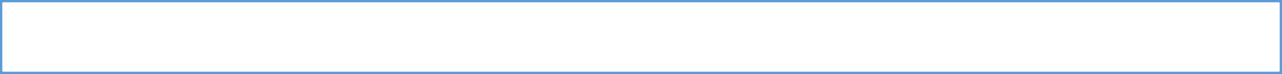


**Matterial para enseñar en una charla:**

1. ¿Por qué es tan importante la planificación familiar?

De acuerdo con un reportaje llamado “Guatemala Health System Assessment 2015: Private Sector Assessment of Family Planning, Antenatal Care, and Delivery”, Guatemala tiene la tasa de mortalidad más alta en todo Centroamérica con los niños menores de 5 años (Cisek et al.). Como resultado de malnutrición y problemas económicos, los niños puedan sufrir. Por eso, es necesario que mujeres tengan la oportunidad y habilidad de planear los bebes. Hay muchas maneras en las que las mujeres pueden planear los bebés. Con la planificación familiar, las mujeres pueden asegurar que los nenos no sufran.

1. ¿Cuáles son los mitos sobre la planificación familiar?

## MITO #1: Los anticonceptivos causan abortos.

- La verdad: Los anticonceptivos impiden 112 millón abortos cada año. Si las mujeres usan los anticonceptivos, ellas pueden controlar los embarazos. Por eso, menos mujeres ejecutan abortos porque menos embarazos inesperados

ocurren (“Get Real: The Facts vs. Myths on Population, Family Planning, and Reproductive Health”, *Mulat Pinoy*). Además, los métodos de planificación familiar no matan al bebé. Todos los métodos funcionan de una manera diferente. Sin embargo, ningún método mata al bebé. Todos los métodos (excepto la T de cobre) impiden la fecundación. La T de cobre impiden la implantación (Carey, “Which Birth Control is Right for You?”).

## MITO #2: Los anticonceptivos son peligrosos y pueden causar cáncer.

- La verdad: De hecho, los anticonceptivos pueden bajar el riesgo de cáncer. Por ejemplo, hubo un estudio con 46,000 mujeres que usaron las pastillas anticonceptivas. El estudio duró por 39 años. Los investigadores encontraron que las mujeres que tomaron las pastillas tuvieron una tasa baja del cáncer en comparación con las mujeres que no tomaron las pastillas (“Get Real: The Facts vs. Myths on Population, Family Planning, and Reproductive Health”, *Mulat Pinoy*). Muchos estudios han probado que los métodos anticonceptivos son seguros y beneficiosos para la mujer.

## MITO #3: Los anticonceptivos causan la infertilidad.

- La verdad: Muchos estudios han probado que los anticonceptivos no causan la infertilidad (Mikkelsen et al., “Pre-­gravid Oral Contracteptive Use and Time to Pregnancy: A Danish Prospective Cohort Study”). Es posible que el ciclo menstrual sea irregular por algunos meses después del uso de los anticonceptivos, pero el ciclo menstrual regresará a normal después de un tiempo corto.

## MITO #4: No se puede planificar de una manera natural.

- La verdad: Hay muchas opciones. Si una mujer entiende el ciclo de ovulación y el método de retiro, ella puede planificar. Este método no es el más efectivo, pero puede evitar 96% de los embarazos si una mujer y su pareja lo usan correctamente (“Planificación Familiar”, *Organización Mundial De La Salud*).

## MITO #5: Los anticonceptivos no funcionan.

- La verdad: La tasa de eficacia de la mayoría de los métodos es más de 95%. Sin embargo, si una mujer no usa el método correctamente, el método no funcionará. Por ejemplo, si una mujer usa las inyecciones cada 3 meses, ella tiene que recibir las inyecciones cada 3 meses. Si ella no lo hace, ella puede quedar embarazada. La tasa de eficacia baja cuando las mujeres no usan los métodos eficazmente (“Planificación Familiar”, *Organización Mundial De La Salud*). Es importante que las mujeres hablen con los médicos sobre la manera correcta para evitar el embarazo.

1. Los efectos secundarios

- Es común tener algunos efectos secundarios. Sin embargo, la mayoría de estos efectos desaparecen después del cuerpo se adapta al anticonceptivo. Además,

todos los cuerpos son diferentes. Por eso, es necesario que la mujer hable con el medico sobre el mejor método para ella. Algunas veces, las mujeres cambian el método antes de encontrar la opción perfecta. Como todos los medicamentos, los anticonceptivos pueden causar efectos secundarios. Hay una lista de todos los efectos secundarios (con cada método) debajo de número 4.

1. Los métodos de la planificación familiar

| **Método** | **Descripción** | **Cómo funciona** | **Eficacia para prevenir el**  **embarazo** | **Observaciones/Efectos Secundarios** |
| --- | --- | --- | --- | --- |
| Las pastillas | Contiene dos hormonas (estrógeno y progestágeno) | Evita la liberación de óvulos por los ovarios (ovulación) | >99% si se usa de manera correcta y sostenida | Disminuye el riesgo de cáncer endometrial y ovárico |
| Implantes | Cilindros o cápsulas pequeños y flexibles que se colocan debajo de la piel del brazo;; contienen únicamente progestágeno | Hace más espeso el moco del conducto del cuello uterino, lo que impide el encuentro de los espermatozoides con el óvulo y evita la ovulación | >99% | Debe ser insertado y extraído por personal sanitario;; se puede usar de 3 a 5 años, según el tipo;; las hemorragias vaginales irregulares son comunes pero no dañinas. |
| Inyecciones | Se inyectan cada mes o cada 3 meses por vía intramuscular;; contienen estrógeno y  progestágeno | Impide que los ovarios liberen óvulos (ovulación) | >99% si se usan de manera correcta y sostenida | Las hemorragias vaginales irregulares son comunes pero no dañinas. |
| Dispositivo intrauterino (DIU)  -­ La T de cobre | Dispositivo plástico flexible y pequeño que contiene un asa o cubierta de cobre y se | El cobre daña los espermatozoides e impide que se junten con el óvulo | >99% | Disminuye la frecuencia de cólico menstrual y los síntomas de endometriosis;; amenorrea (ausencia de hemorragia menstrual) puede ocurrir |

|  | inserta en el útero |  |  |  |
| --- | --- | --- | --- | --- |
| Condón masculino | Cubierta que envuelve el pene erecto | Forma una barrera que impide el encuentro de los espermatozoides  con el óvulo | 98% si se usa de manera correcta y sostenida | También protege de las infecciones de transmisión sexual, en particular la causada por el VIH |
| Condón femenino | Forro que se adapta holgadamente a la vagina;; está hecho de un material plástico transparente,  fino y suave | Forma una barrera que impide el encuentro de los espermatozoides con el óvulo | 90% si se usa de manera correcta y sostenida | También protege de las infecciones de transmisión sexual, en particular la causada por el VIH |
| Esterilización masculina (vasectomía) | Anticoncepción permanente por la cual se bloquean o cortan los tubos (conductos deferentes) que transportan los espermatozoides desde los  testículos | Impide que haya espermatozoides en el semen eyaculado | >99%  después de la evaluación del semen a los 3 meses | No afecta el funcionamiento sexual del hombre |
| Esterilización femenina (ligadura de las trompas) | Anticoncepción permanente por la cual se bloquean o cortan las trompas de  Falopio | Los óvulos no pueden juntarse con los espermatozoides | >99% | Es fundamental que la mujer tenga el conocimiento de causa |
| Método de la  amenorrea del amamantamiento | Es un método de  anticoncepción temporal para las | Impide que los ovarios liberen | 99% si se  aplica de manera | Es un método natural |

|  | mujeres recién | óvulos | correcta y |  |
| --- | --- | --- | --- | --- |
|  | paridas que no | (ovulación) | sostenida |  |
|  | han vuelto a |  |  |  |
|  | menstruar;; exige |  |  |  |
|  | el |  |  |  |
|  | amamantamiento |  |  |  |
|  | exclusivo o día y |  |  |  |
|  | noche |  |  |  |
|  | completos, de |  |  |  |
|  | una criatura |  |  |  |
|  | menor de 6 |  |  |  |
|  | meses |  |  |  |
| Píldoras | Son pastillas que | Retrasa la | 1/100 | Es un método para usar |
| anticonceptivas | se toman para | ovulación | mujeres | después de la relación |
| de emergencia | prevenir el |  | quede | sexual |
|  | embarazo hasta |  | embarazada |  |
|  | 5 días después |  | después de |  |
|  | de una relación |  | que ella |  |
|  | sexual sin |  | tome la |  |
|  | protección |  | pastilla |  |
| Método de los | Este método | Impide el | 86% | Es difícil de aplicar si |
| días | consiste en | embarazo, si se |  | hay infección vaginal u |
|  | determinar los | evita el coito sin |  | otra infección que altere |
|  | períodos fértiles, | protección |  | el moco cervical. Es un |
|  | prestando | durante los días |  | método natural. |
|  | atención a la | fértiles |  |  |
|  | presencia de |  |  |  |
|  | moco cervical (si |  |  |  |
|  | hay secreciones, |  |  |  |
|  | tipo, color y |  |  |  |
|  | consistencia) |  |  |  |
| Método del | Consiste en | Se impide el | 75% | Es un método natural |
| collar/ritmos | observar el ciclo | embarazo |  |  |
|  | menstrual | evitando el coito |  |  |
|  | durante 6 meses, | sin protección |  |  |
|  | restar 18 de la | durante el |  |  |
|  | duración del ciclo | posible día fértil |  |  |
|  | más corto | y el posible |  |  |
|  | (primer día fértil | último día fértil |  |  |
|  | estimado) y |  |  |  |

|  | restar 11 de la duración del ciclo más largo (último día fértil  estimado) |  |  |  |
| --- | --- | --- | --- | --- |
| Coitus | Consiste en | Se trata de | 96% si se | Es difícil determinar |
| interruptus/retiro | retirar el pene de | impedir que la | usa de | correctamente cuando |
|  | la vagina antes | esperma entre | forma | hay que retirar. Es un |
|  | de la eyaculación | en la vagina | correcta y | método natural. |
|  | y eyacular fuera | para evitar la | sostenida. |  |
|  | de esta, | fecundación | 73% tal y |  |
|  | asegurándose de |  | como se |  |
|  | que el semen no |  | practica |  |
|  | entre en contacto |  | comúnmente |  |
|  | con los genitales |  |  |  |
|  | externos |  |  |  |

(“Planificación Familiar”, *Organización Mundial De La Salud*)

1. Las ofertas en Primeros Pasos

- La T de cobre
- Algunas veces: condones, implantes, pastillas e inyecciones (depende del tiempo y las donaciones)

1. Los lugares donde las mujeres puedan obtener la planificación familiar (además de Primeros Pasos)

- Clínica de Centro de Comunitario en Tierra Colorada Baja
- Clínica de Centro de Comunitario en Xecaracoj
- Clínica de Centro de Comunitario en Llano de Pinal
- Centro de Salud en Xela
- Clínica de Centro de Comunitario en Tierra Colorada Alta
- Clínica de Centro de Salud en Las Majadas
- Clínica de Centro de Salud en Chuicavioc
- Clínica de Centro de Salud en Candelaria

(De acuerdo con el trabajo voluntario de Arielle Clemens en Primeros Pasos)

Links a la información:

- https:[//www.usaid.gov/sites/default/files/documents/1862/Guatemala-­](http://www.usaid.gov/sites/default/files/documents/1862/Guatemala-)SHOPS-­ PSA-­full-­report-­English-­12-­16-­15.pdf
- [http://bixby.berkeley.edu/wp-­](http://bixby.berkeley.edu/wp-)content/uploads/2011/04/The-­Facts-­vs.-­Myths-­on-­ Population-­Family-­Planning-­and-­Reproductive-­Health.pdf
- [http://www.healthline.com/health/birth-­](http://www.healthline.com/health/birth-)control/methods#prescription2
- https://academic.oup.com/humrep/article/28/5/1398/940795/Pre-­gravid-­oral-­ contraceptive-­use-­and-­time-­to
- <http://www.who.int/mediacentre/factsheets/fs351/es/>

# Las enfermedades transmitidas por relaciones sexuales

## Las estadísticas de nuestra encuesta:

- 35% de las mujeres no han oído de las enfermedades transmitidas por relaciones sexuales
- 27% de las mujeres no han oído de la enfermedad VIH
- 18% de las mujeres no han oído del Sida


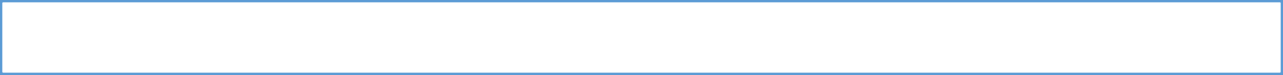


**Matterial para enseñar en una charla:**

1. ¿Qué son las enfermedades de transmisión sexual?

Algunas veces, se refieren a estas enfermedades como enfermedades venéreas. Son un grupo de enfermedades causadas por diversos agentes infecciosos que se adquieren por la actividad sexual. La tasa de estas enfermedades está aumentando en parte debido a más actividad sexual de las personas jóvenes. Además, muchas de estas enfermedades no producen síntomas, especialmente en las mujeres. Sin embargo, la mujer (sin síntomas) puede transmitir la enfermedad a su pareja sexual (“Enfermedades De Transmisión Sexual”, *Inicio*). Los doctores pueden curar la mayoría de estas enfermedades, pero es necesario que las mujeres sepan los síntomas para curarlas.

1. ¿Cuáles son los tipos diferentes (y los síntomas)?

- Virus Immunodeficiencia Humana (VIH)
  - Puede infectar a una persona por el contacto directo con líquidos corporales o sangre infectada (la vía más común de transmisión VIH es la sexual)
  - La enfermedad se convierte en el Sida después de 5-­8 años. Sin embargo, si una persona tiene tratamiento, el Sida puede llegar 20 años después de la infección inicial.
  - Los síntomas empiezan una semana después de la infección inicial. No obstante, muchas personas no tienen síntomas. Los síntomas que pueden aparecer incluir: dolor de garganta, fiebre, cansancio y dolor de cabeza. Duran por 14 días.
  - Después de muchos años, el virus sigue replicándose dentro de las células del sistema inmune. Poco a poco, irán disminuyendo las defensas. Los síntomas de esta etapa pueden incluir la pérdida de peso, lesiones cutáneas y tumores.
  - El tratamiento: No se puede curar la enfermedad. Sin embargo, los medios se pueden emplear una combinación de varios fármacos antirretrovirales.
- Herpes genital
  - Puede infectar a una persona a través del sexo (el virus puede causar úlceras genitales)
  - Con el primer episodio: Los síntomas duran por 15 días. Incluyen múltiples lesiones dolorosas con contenido líquido en su interior en la zona genital y el picor.
  - Con episodios no primarios: La persona ya tiene anticuerpos. Como resultado, los síntomas son más leves que el primer episodio.
  - El tratamiento: medicamento (llamado Aciclovir)
- Virus del Papiloma Humano (VPH)
  - Hay muchísimos tipos de VPH. Hay algunos de bajo riesgo y de alto riesgo. Los más frecuentes son asintomáticas.
  - Los tipos con alto riesgo tienen estos síntomas: verrugas localizadas en los genitales (dolorosas y causan picor)
  - El tratamiento: crioterapia (con nitrógeno líquido). Se puede impedir estas enfermedades con una vacuna llamada Gardasil (para las mujeres de mayor edad). Además, los tipos de bajo riesgo se resuelven espontáneamente.
- Gonorrea
  - Se infecta a una persona por la bacteria *Neisseria gonorrhoeae*.
  - Los síntomas aparecen después de 2-­5 días. Estos incluyen: vaginal amarillenta, dolor al orinar, sangrado inter-­menstrual y dolor abdominal bajo. Si una persona no recibe el tratamiento necesario, la infección puede causar la enfermedad inflamatoria pélvica (lo que pueda causar la infertilidad).
  - El tratamiento: Una dosis intramuscular de ceftriaxona y doxiciclina
- Sífilis
  - Se infecta a una persona por la bacteria *Treponema pallidum*. Esta enfermedad tiene periodos de latencia en los que la enfermedad solo es detectable mediante pruebas de sangre.
  - Los síntomas: Al principio, una lesión (el chancro) aparece en los genitales externos o en la vagina. Después de un tiempo de 6 semanas-­6 meses, una erupción cutánea generalizada sin picor aparece (en las palmas de las manos y a las plantas de los pies). Si todavía no ha recibido el tratamiento, sífilis terciaria ocurre. Esta forma incluye afectación vascular y afectación neurológica (neurosífilis).
  - El tratamiento: la penicilina G
- Clamidia
  - Se infecta a una persona por la bacteria *Chlamydia trachomatis*.
  - Los síntomas de las mujeres: flujo vaginal anormal (puede tener un fuerte olor), sensación de ardor al orinar, dolor durante las relaciones sexuales. Los síntomas de los hombres: secreción del pene, sensación de ardor al orinar, picazón alrededor de la abertura del pene y dolor e inflamación en uno o ambos testículos
  - El tratamiento: doxiciclina

(Alves, “Enfermedades De Transmisión Sexual”)

1. ¿Cómo se puede proteger si mismo de estas enfermedades?

- Tener relaciones sexuales con una pareja no infectada
- Siempre utilice los condones

Links a la información:

- https:[//www.uhclatino.com/content/lat-­](http://www.uhclatino.com/content/lat-)muhclati/uhc-­latino/es/mantenerse-­ saludable/consejos-­de-­salud-­al-­instante/consejos-­de-­salud/enfermedades-­de-­ transmision-­sexual.html
- [http://www.webconsultas.com/enfermedades-­](http://www.webconsultas.com/enfermedades-)de-­transmision-­sexual/virus-­ inmunodeficiencia-­humana-­vih-­518

# El cáncer de la matriz

## Las estadísticas de nuestra encuesta:

- 30% de las mujeres no han oído de cáncer de la matriz
- Sin embargo, 30% de las mujeres que no supieron sobre el cáncer de la matriz han tenido un Papanicolaou en el pasado. Es posible que ellas no sepan el propósito de un Papanicolaou.


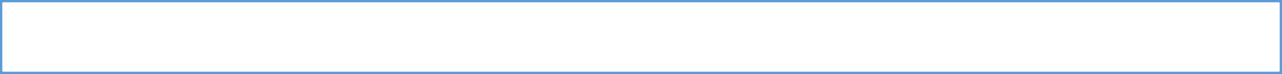


**Matterial para enseñar en una charla:**

1. ¿Qué es cáncer de la matriz?

El cérvix (o cuello uterino) es la parte inferior del útero que forma el canal que lleva a la vagina. En este tipo de cáncer, la mayor parte de los tumores surgen en el lugar donde se une el ectrocérvix con el endocérvix. Las células normales del cérvix empiezan a transformarse y crecen de una manera descontrolada.

Este cáncer es más común en mujeres que tienen entre 40 y 55 años. El virus del papiloma humano (VPH) (OJO: arriba) puede causar el cáncer de la matriz. De hecho, el VPH está presente en el 99% de los casos de cáncer de cérvix. El consumo de tabaco y la promiscuidad sexual pueden causar este cáncer también. Sin embargo, este cáncer puede desarrollar sin estas causas. Por eso, es necesario monitorear la salud de la matriz (“Cáncer De Cuello De útero: Toda La Información”, *CuidatePlus*).

1. ¿Cómo podemos proteger a las mujeres?

Podemos proteger a las mujeres con la monitorización de la matriz. Las pruebas de Papanicolaou pueden detectar hasta un 90% de los cánceres de cérvix. Además, estas prueban pueden detectarlo antes de que aparezcan los síntomas. Es recomendable que las mujeres se hagan una prueba de Papanicolaou cada año cuando comienzan a ser sexualmente activas (“Cáncer De Cuello De útero: Toda La Información”, *CuidatePlus*). Cuando una prueba de Papanicolaou detecta el cáncer durante una etapa temprana, la tasa de sobrevida es 91% (“Cervical Cancer: Statistics.”, *Cancer.Net*).

Links a la información:

- [http://www.cuidateplus.com/enfermedades/cancer/cancer-­](http://www.cuidateplus.com/enfermedades/cancer/cancer-)cuello-­utero.html
- [http://www.cancer.net/cancer-­](http://www.cancer.net/cancer-)types/cervical-­cancer/statistics

# Referencias

"Cáncer De Cuello De útero: Toda La Información." CuidatePlus. N.p., 29 Nov. 2016.

Web. 06 July 2017.

"Cervical Cancer: Statistics." Cancer.Net. N.p., 16 Mar. 2017. Web. 06 July 2017. "Cesarean Section -­ Risks and Complications." WebMD. WebMD, n.d. Web. 05 July

2017.

"Delivery Care." UNICEF DATA. N.p., n.d. Web. 05 July 2017. "Enfermedades De Transmisión Sexual." Inicio. N.p., n.d. Web. 06 July 2017.

"Fertility & Ovulation Pictures: Facts to Help You Get Pregnant."WebMD. WebMD, 8 Mar. 2017. Web. 05 July 2017.

"Get Real: The Facts vs. Myths on Population, Family Planning, and Reproductive Health." Mulat Pinoy (n.d.): n. pag. Web. 6 July 2017.

"Planificación Familiar." Organización Mundial De La Salud. World Health Organization, Dec. 2016. Web. 06 July 2017.

"Signs and Symptoms of Ovulation." American Pregnancy Association. N.p., 01 Sept.

2016. Web. 05 July 2017.

"WHO Statement on Caesarean Section Rates." World Health Organization. World Health Organization, n.d. Web. 05 July 2017.

Alves, Silvia Chacón. "Enfermedades De Transmisión Sexual."Webconsultas. N.p., 25 Apr. 2017. Web. 06 July 2017.

Betrán, Ana Pilar, Jianfeng Ye, Anne-­Beth Moller, Jun Zhang, A. Metin Gülmezoglu, and Maria Regina Torloni. "The Increasing Trend in Caesarean Section Rates: Global, Regional and National Estimates: 1990-­2014." PLoS ONE. Public Library of Science, 5 Feb. 2016. Web. 05 July 2017.

Carey, Elea. "Which Birth Control Is Right for You?" Healthline. Healthline Media, 30 July 2015. Web. 06 July 2017.

Cisek, Cindy, Chloé Revuz, and Christina Kramer. "Guatemala Health System

Assessment 2015: Private Sector Assessment of Family Planning, Antenatal Care, and Delivery." SHOPS (n.d.): 24. USAID. Dec. 2015. Web. 6 July 2017.

Khazan, Olga. "Why Most Brazilian Women Get C-­Sections." The Atlantic. N.p., 14 Apr.

2014. Web. 5 July 2017.

Mikkelsen, Ellen M., Anders H. Riis, Lauren A. Wise, Elizabeth E. Hatch, Kenneth J. Rothman, and Henrik Toft Sørensen. "Pre-­gravid Oral Contraceptive Use and Time to Pregnancy: A Danish Prospective Cohort Study." Human Reproduction. Oxford University Press, 20 Feb. 2013. Web. 06 July 2017.
